# Cavotricuspid Isthmus Line with a Variable-loop Biphasic Circular Pulsed Field Ablation Catheter Integrated with a 3-dimensional Mapping System - A Case Report

**DOI:** 10.2174/011573403X401914250922111014

**Published:** 2025-10-09

**Authors:** Panteleimon E. Papakonstantinou, Peter Murray, Jim O’Brien

**Affiliations:** 1 Heart and Vascular Centre and Cardiovascular Research Institute Dublin, Royal College of Surgeons of Ireland, Mater Private Hospital, 71 Eccles St, D07 WKW8, Dublin, Ireland

**Keywords:** Cavotricuspid isthmus, pulsed field ablation, atrial fibrillation, atrial flutter, catheter ablation, antiarrhythmic therapy

## Abstract

**Introduction:**

Pulsed field ablation (PFA) minimizes the risk of esophageal injury, pulmonary vein stenosis, and permanent phrenic nerve damage. However, when PFA is applied near coronary arteries, it has been associated with the induction of coronary vasospasm, which is typically asymptomatic but occasionally results in ventricular arrhythmias. Pre-treatment strategy with intravenous nitroglycerin can significantly reduce the occurrence of this complication.

**Case Presentation:**

A 79-year-old male with cavotricuspid isthmus (CTI)-dependent atrial flutter and atrial fibrillation underwent catheter ablation after antiarrhythmic therapy failure. Following pulmonary vein and posterior wall isolation using a novel variable-loop biphasic circular pulsed field ablation (PFA) catheter integrated with a 3D mapping system (VARIPULSE catheter, CARTO mapping system, Johnson and Johnson, USA), CTI ablation was performed with the same system. Twenty-one applications resulted in sinus rhythm restoration and confirmed bidirectional block, with no complications.

**Conclusion:**

To our knowledge, this is the first real-world reported case of CTI-dependent atrial flutter successfully treated with the mapping-integrated PFA system, with clinical and rhythm follow-up extending to 6 months. Further studies are needed to assess its role in non-pulmonary vein ablation.

## 1 INTRODUCTION

Pulsed field ablation (PFA) operates through a distinct mechanism that minimizes the risk of esophageal injury, pulmonary vein stenosis, and permanent phrenic nerve damage. Nonetheless, when PFA is applied near coronary arteries, it has been associated with the induction of coronary vasospasm [1, 2], which is typically asymptomatic but occasionally results in ventricular arrhythmias [3]. Pre-treatment strategy with intravenous (IV) nitroglycerin (NTG) can significantly reduce the occurrence of this complication [4, 5].

The VARIPULSE^TM^ catheter is a novel variable-loop biphasic circular PFA catheter that integrates with the CARTO^TM^ 3D electroanatomic mapping system. This allows precise anatomical visualization and lesion delivery within complex atrial anatomies. While PFA is currently approved for pulmonary vein isolation (PVI), its off-label use in non-PV targets, such as the cavotricuspid isthmus (CTI), remains under investigation.

We have, herein, reported a case of PFA cavotricuspid isthmus (CTI) line, which was performed with a variable-loop biphasic circular pulsed field ablation catheter integrated with a 3-dimensional mapping system after IV NTG infusion.

## 2 CASE PRESENTATION

A 79-year-old male with a history of cavotricuspid isthmus (CTI)-dependent atrial flutter (Figs. **1A** and **1B**) and atrial fibrillation presented for catheter ablation after failure of antiarrhythmic therapy. His medical history included coronary artery disease (coronary artery bypass surgery), tissue aortic valve replacement, dyslipidemia, depression, chronic lymphocytic leukemia, and benign prostatic hyperplasia. His regular medications included apixaban 5 mg, bisoprolol 2.5 mg, amiodarone 200 mg, atorvastatin 40 mg, sertraline 150 mg, tamsulosin 400 mcg, acalabrutinib 100 mg, and famciclovir 250 mg.

Under general anesthesia, venous access was obtained, and a decapolar catheter was positioned in the coronary sinus. Initial electroanatomic mapping revealed atrial flutter activating from the coronary sinus (CS) 9,10 to CS 1,2 with a cycle length of 340 ms. Following systemic heparinization and direct transseptal puncture using a medium curl bi-directional guiding sheath (CARTO VIZIGO™, Johnson and Johnson, USA), bilateral pulmonary vein isolation and posterior wall isolation were performed using a variable-loop biphasic circular pulsed field ablation catheter integrated with a 3-dimensional mapping system (VARIPULSE^TM^ catheter, CARTO^TM^ mapping system, Johnson and Johnson, USA). Left atrium mapping revealed no significant scar and normal pulmonary vein anatomy. Attention was then turned to the right atrium, where CTI-dependent flutter was confirmed *via* CARTO^TM^ activation mapping, showing the full flutter cycle length (Figs. **2** and **3**). The His bundle was carefully mapped.

Ablation was performed along the CTI using the VARIPULSE^TM^ catheter (Fig. **4**). A total of 21 applications were delivered, with 3 applications per ablation site, covering 7 contiguous sites along the CTI (Fig. **5**). This approach was based on operator experience and prior protocols used in pulmonary vein ablation with this catheter system. IV NTG was administered to deter coronary spasm, using an NTG pre-treatment strategy (2 mg initial bolus, with immediate commencement of IV NTG infusion at 0.5 mg/hr, 2 minutes prior to commencing ablation, and titration every 1-2 minutes 0.5 mg/hr during the ablation). Spontaneous reversion to normal sinus rhythm occurred during ablation. The total duration of the CTI line ablation was 13 minutes.

Post-ablation remapping, performed immediately after lesion delivery, complete bidirectional block was confirmed (Fig. **6**). Table **1** summarizes the key procedural parameters. No ST-segment elevation, arrhythmias, or other procedural complications were observed during or after ablation. Amiodarone was discontinued after the ablation procedure. At 6-month follow-up, the patient did not experience symptom recurrence and was in sinus rhythm on 12-lead ECG (Fig. **1B**).

## 3 DISCUSSION

PFA has been proven to be efficient and safe for pulmonary vein isolation [5-7]. However, the off-label use of PFA for non-pulmonary vein ablation, including the CTI line, has been reported in the literature [4, 5, 7-9]. In particular, CTI ablation using a pentaspline PFA catheter has been proven efficient and safe in a small series of patients [10]. However, PFA *via* a pentaspline catheter of the cavotricuspid isthmus (CTI) and mitral isthmus (MI) has been linked to acute coronary artery spasm and mild luminal narrowing at three months. While intracoronary vasodilator therapy may help prevent or manage acute spasm, the potential for arterial stenosis persists, underscoring the need for careful monitoring and long-term follow-up [11].

Lesion durability is a critical concern. While radiofrequency ablation and cryoablation have been the standard for CTI ablation, PFA may offer benefits in lesion selectivity and reduced collateral damage. However, long-term comparative data are still lacking. This novel mapping integrated PFA system (VARIPULSE^TM^ catheter, CARTO^TM^ mapping system, Johnson and Johnson, USA) proved its safety and effectiveness for pulmonary vein isolation during a 12-month follow-up period in the inspIRE study [6].

The patient had a history of coronary artery bypass grafting (CABG), which was clinically stable with no recent angina symptoms. No new coronary imaging was performed peri-procedurally, as no ST-segment changes or arrhythmias occurred. We recommend a structured follow-up that includes a 12-lead ECG at 1 and 6 months, 24-hour Holter monitoring at 6-12 months, and echocardiography at 6-12 months to assess rhythm outcomes and structural cardiac status.

## CONCLUSION

To our knowledge, this is the first real-world report in humans describing the CTI-dependent atrial flutter management *via* this novel variable-loop biphasic circular pulsed field ablation catheter integrated with a 3-dimensional mapping system, with clinical and rhythm follow-up extending to 6 months. Establishment of NTG IV infusion protocols could be useful for the implementation of PFA in areas close to the coronary arteries. The long-term effectiveness and durability of lesions created *via* this catheter for non-pulmonary vein ablation should be tested in future studies.

## Figures and Tables

**Fig. (1):**
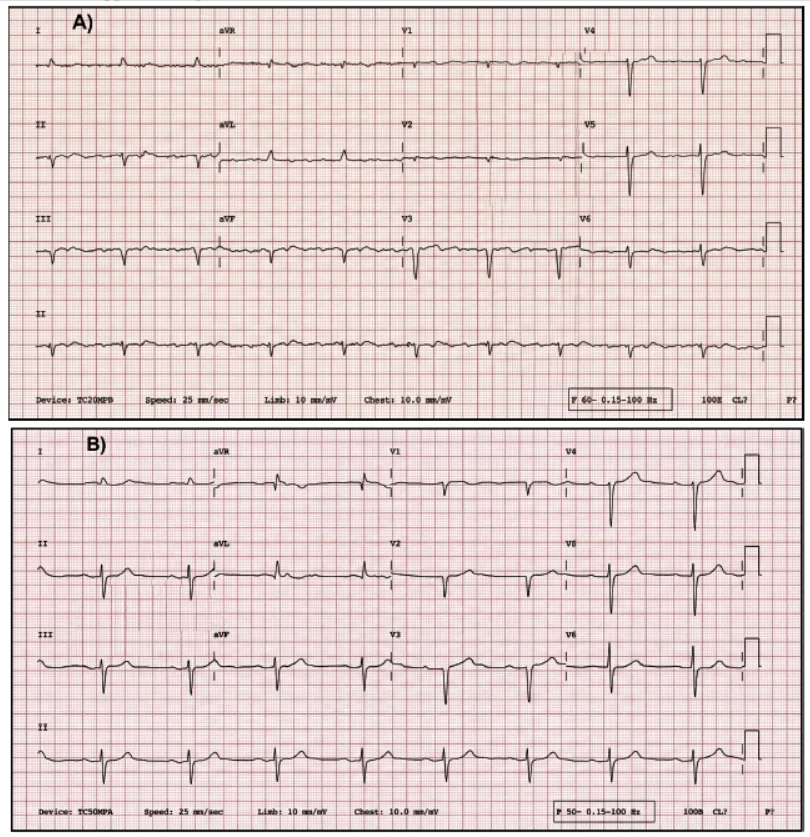
**A.** 12-lead ECG of the CTI-dependent atrial flutter. **B.** Post-CTI line ablation 12-lead ECG showing normal sinus rhythm after a 6-month follow-up period.

**Fig. (2):**
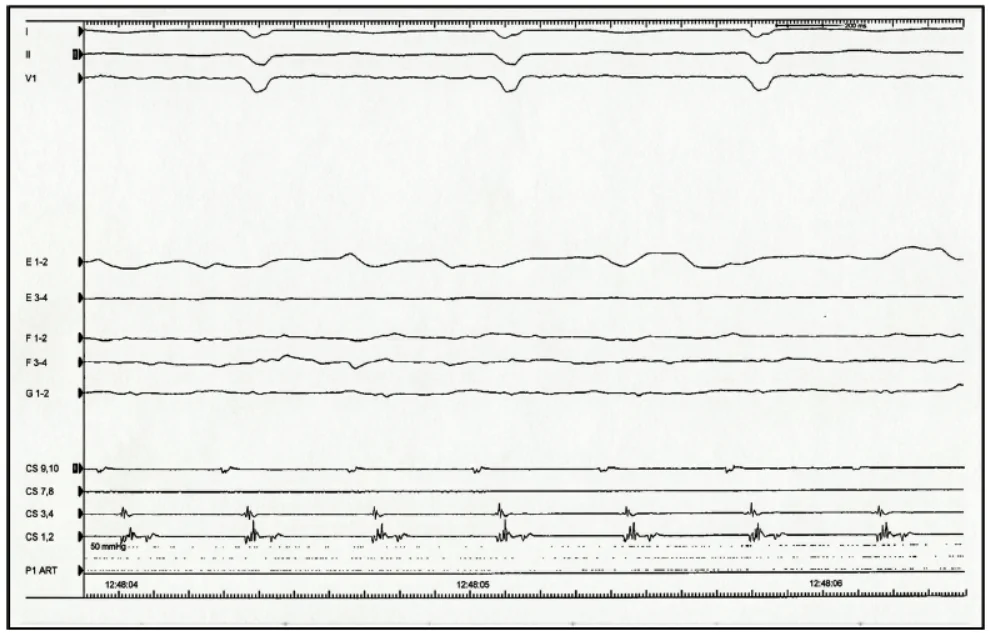
Intracardiac electrograms showing atrial flutter. A decapolar coronary sinus (CS) catheter was placed with CS 1-2 at the lateral mitral annulus and CS 9-10 at the CS ostium. Atrial activation followed a proximal-to-distal pattern (CS 9-10 to CS 1-2).

**Fig. (3):**
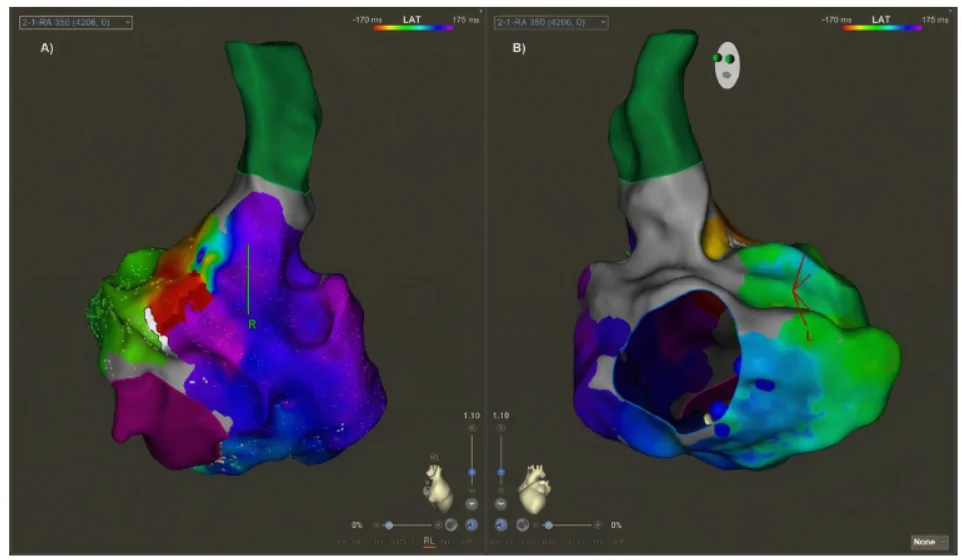
Electroanatomic map of the right atrium showing a CTI-dependent atrial flutter. **A.** Right lateral view. **B.** Left anterior oblique (LAO) view.

**Fig. (4):**
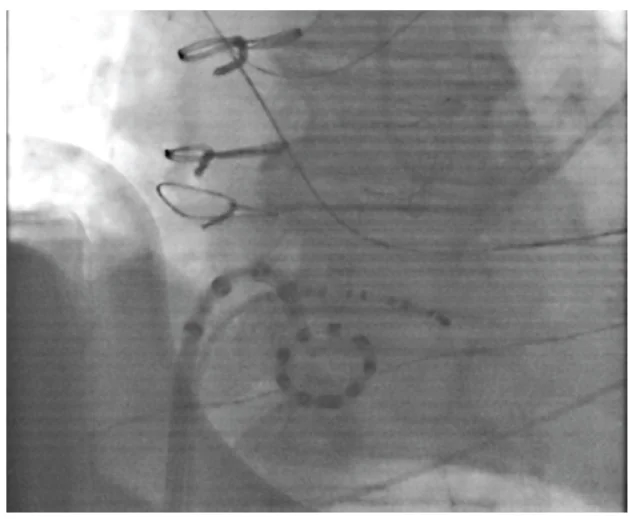
Fluoroscopic anterior-posterior view showing the VARIPUSE^TM^ catheter positioned at the cavotricuspid isthmus (CTI) line.

**Fig. (5):**
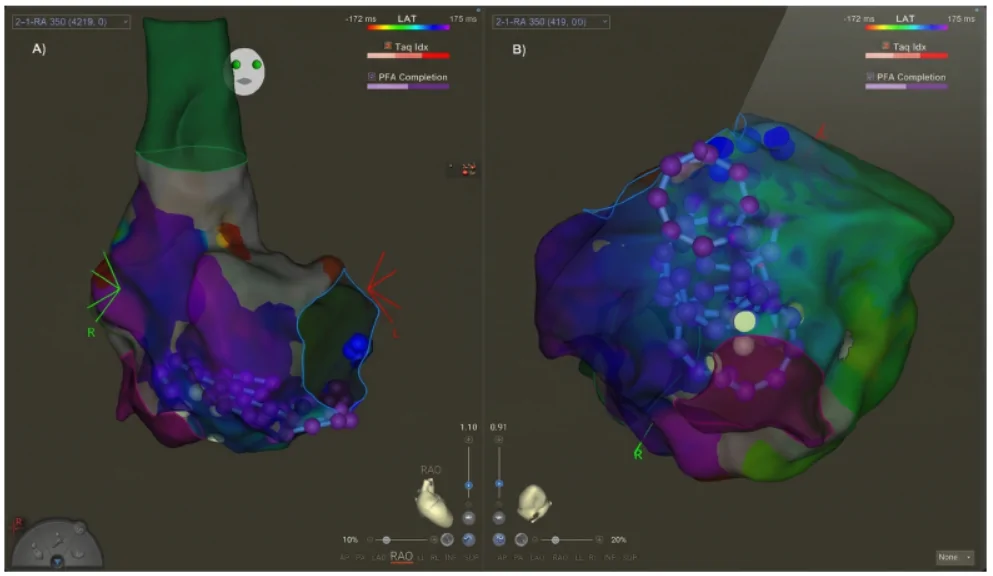
Electroanatomic map of the right atrium showing seven ablations on the CTI line. One ablation was equal to three applications. **A.** Right oblique (RAO) view. **B.** Inferior view.

**Fig. (6):**
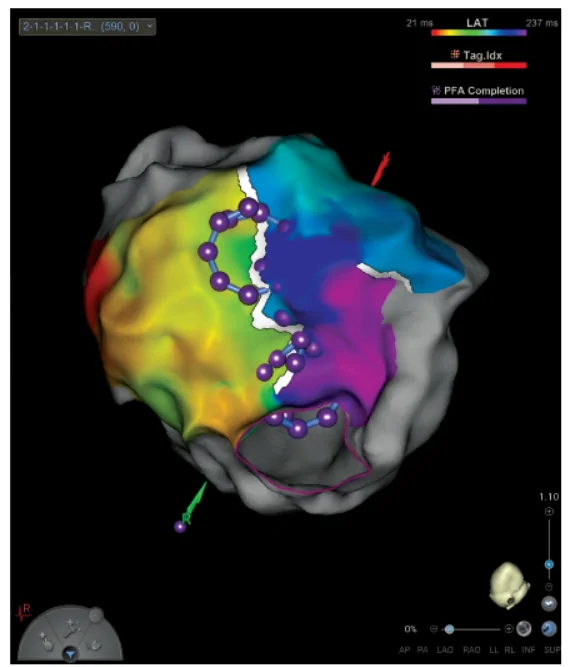
Post-ablation remapping confirming bidirectional block and interruption of the atrial flutter circuit.

**Table 1:** Key procedural parameters.

**Parameter**	**Value**
Total CTI ablation applications	21 (3 per site × 7)
Procedural times	-
Pre-ablation RA time	3 minutes
Total CTI ablation time	10 minutes
Post-ablation mapping time	1 minute
Post-ablation observation	30 minutes
NTG	-
NTG IV bolus	2 mg
NTG infusion	0.5 mg/hr, 2 minutes prior to commencing ablation; titration every 1-2 minutes 0.5 mg/hr during ablation
NTG infusion time	Total infusion time: 17 minutes 2 minutes prior to ablation 10 minutes during the ablation 5 minutes post-ablation

**Abbreviations:** RA: Right atrium; CTI: Cavotricuspid isthmus; NTG: Nitroglycerin

## Data Availability

Not applicable.

## LIST OF ABBREVIATIONS

CABG: Coronary Artery Bypass Grafting
CS: Coronary Sinus
CTI: Cavotricuspid Isthmus
IV: Intravenous
MI: Mitral Isthmus
NTG: Nitroglycerin
PFA: Pulsed Field Ablation
PVI: Pulmonary Vein Isolation

## STANDARDS OF REPORTING

CARE guidelines were followed.
